# Photo(electro)catalyst of Flower-Like Cobalt Oxide Co-Doped g-C_3_N_4_: Degradation of Methylene Blue under Visible Light Illumination

**DOI:** 10.3390/ma15124104

**Published:** 2022-06-09

**Authors:** Qiuhua Li, Qunhui Wang

**Affiliations:** 1 School of Energy and Environmental Engineering, University of Science and Technology Beijing, Beijing 100083, China; wyuchemlqh@126.com; 2 School of Biotechnology and Health Sciences, Wuyi University, Jiangmen 529020, China

**Keywords:** Co(OH)_2_, g-C_3_N_4_, methylene blue dyes, photo(electro)catalyst

## Abstract

This work reported on the solid state synthesis of the flower-like Co(OH)_2_/g-C_3_N_4_ nanocomposite, using a modified hydrothermal method, for the degradation of MB, an organic pollutant. These nanomaterials were characterized for structure, surface morphology and composition using XRD, SEM and XPS, respectively. The photocatalytic activities of the as-prepared materials loaded on FTO glass substrates were evaluated for their degradation of methylene blue (MB) under visible irradiation and constant voltage. The promoting effect of Fw-Co(OH)_2_ on g-C_3_N_4_ was investigated under the influence of introduced various Co(OH)_2_ amounts. The fabricated composite catalyst showed significantly improved catalytic performance compared to pristine g-C_3_N_4_. Degradation by 25% Fw-Co(OH)_2_/g-C_3_N_4_ can achieve about a 100% ratio within 180 min under visible light in a three-electrode system. Moreover, Fw-Co(OH)_2_/g-C_3_N_4_ was easily regenerated and reused, and still possessed good degradation ability. These results suggest that Fw-Co(OH)_2_/g-C_3_N_4_ could be promising for application as a low-cost and high-efficiency catalyst for wastewater treatment and organic pollutant degradation.

## 1. Introduction

Water pollution escape from different dye industries including paper, leather, textiles and plastics, has become a critical problem in the world [1] since the dyes used, even at low concentrations, are toxic and threatening to the aquatic system and human health [2]. Various techniques, such as adsorption, membrane processes, electrochemical treatment, photo decomposition and biological methods, have been developed to remove dyes from wastewater [3,4]. Among these methods, from carbon nanoflakes for selective adsorption of water-soluble cationic dyes (MB), to anionic dyes [5], nanofibrous membranes can be used to enhance ion exchange properties. They are promising candidates for the treatment of dye-laden wastewater [6]. Cellulose/alginate monolithic hydrogel is a highly effective adsorbent for methylene blue (MB) removal [7], and photocatalytic degradation based on semiconductors has attracted increasing attention. This is an approach which leads to the generation of free radicals towards water pollution treatment. Eco-friendly and stable photocatalysts, such as TiO_2_, Fe_2_O_3_, CdS and ZnO, have proven to be suitable photocatalysts for the removal of organic water pollutants [8,9,10]. Noble metal catalysts have low ignition temperatures and high catalytic activity, but they are expensive and easy to deactivate, and thus cannot be widely used in business. Non-metallic catalysts are low in cost, with sufficient activity and excellent stability; recently, the metal-free g-C_3_N_4_ photocatalysts have attracted increasing attention [11].

Compared with other wide band-gap semiconductor materials, metal-free g-C_3_N_4_ photocatalysts have a unique band structure which can enable g-C_3_N_4_ to absorb more visible light. Moreover, such catalysts have better thermal and chemical stability in addition to remarkable electronic properties. Hence, g-C_3_N_4_ semiconductors have been used in various applications, for example, in the purification of contaminated water, CO_2_ reduction, hydrogen evolution, energy storage and in humidity sensors [12]. However, the photocatalytic activity of g-C_3_N_4_ is still low. In order to solve the problem, many methods, including synthesis techniques, nanostructure design and electronic structure modulation have been proposed, in order to improve the photocatalytic performance of g-C_3_N_4_ [13,14,15]. Among them, deposition of efficient alternative semiconductors onto g-C_3_N_4_ has proven to be a feasible and effective measure to enhance its photocatalytic activity and efficiency. Nanocomposites with heterojunctions can effectively enhance the separation of electron-hole pairs and improve charge-separation efficiency, resulting in improved photocatalytic performance [16]. Liu et al. [17] prepared g-C_3_N_4_ nanosheets coupled with co-catalyst platinum (Pt), and used them for water splitting. Patnaik et al. [18] summarized the recent significant advances in designing Ag-modified g-C_3_N_4_-based nanocomposites in order to promote their photocatalytic activity.

Cobalt hydroxide (Co(OH)_2_) is attracting increasing attention due to its low cost, earth abundance and unique redox property. Co(OH)_2_ has two types of crystal structure, the α and β phase. Compared to β phase, α phase has a layered structure with relatively larger interlayer spacing, which could enhance the diffusion of ions and become beneficial for electrochemical applications. Its relatively open structure enables rapid diffusion of reactants or products and rapid proton-coupled electron transfer, which make the catalytic active sites easily accessible; this results in high catalytic activity [19,20,21]. Recently, it has been reported that layers of flower-like Co(OH)_2_ assembled with numerous of nanosheets possess excellent structural stability, in addition to optical, electrochemical, electromagnetic adsorptional properties and high selectivities, leading to great potential in for use in water oxidation, supercapacitors, and microwave adsorption materials [22,23,24]. However, to the best of our knowledge, no published papers exist about transition-metal hydroxides.

In order to enhance the photo(electro)catalytic degradation effect, we are going to begin research on the synthesis of Co(OH)_2_-decorated g-C_3_N_4_ heterojunction composite (Fw-Co(OH)_2_/g-C_3_N_4_), and also plan to develop different techniques to study the ability of the synthetic material (Fw-Co(OH)_2_/g-C_3_N_4_) to remove methylene blue (MB) from waste water via its photo(electro)catalytic action.

## 2. Materials and Methods

### 2.1. Materials

Cobalt(II) nitrate hexahydrate (≥99.0%), Cobalt(II) acetate tetrahydrate (≥99.5%), urea (≥99%), Methanol (≥99.5%), Polyvinylpyrrolidone (PVP, ≥98.0%, Shanghai Maclean Co., LTD, Shanghai, China) and 5,5-dimethyl-1-pyrroline-N-oxide (DMPO; ≥97.0%). All the other chemicals were purchased from Guangzhou Chemical Reagent Co., LTD, Guangzhou, China. All chemicals are analytical grade, and used as received without further purification.

### 2.2. Preparation of Film Electrodes

**Figure 1:**
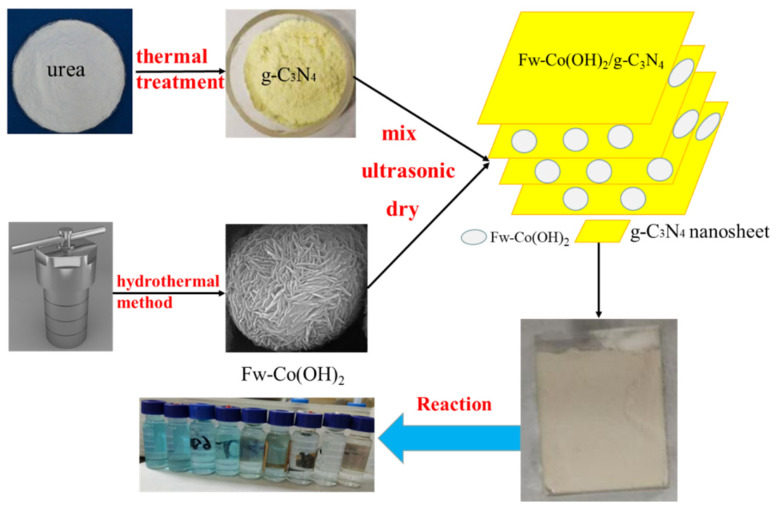
Schematic diagram of the synthesis process.

The g-C_3_N_4_ semiconductor was obtained by thermal treatment. In a conventional process, 5 g of urea as precursor was put into a crucible and calcined at 550 °C for 2 h in air. Flower-like Co(OH)_2_ was synthesized using a modified hydrothermal method. First, 0.498 g of Co(CH_3_COO)_2_·4H_2_O, 0.24 g of urea and 0.5 g of polyvinylpyrrolidone (PVP, K30) were precisely added and dissolved in 40 mL of methanol. After 20 min of ultrasonication, the aqueous solutions were transferred into a 120-milliliter Teflon-steeled autoclave and heated at 200 °C for 6 h in an oven. The pink precipitate was separated by centrifugation, repeatedly rinsed with water and ethanol several times, and then dried at 60 °C under vacuum; the final product was Fw-Co(OH)_2_. The synthesized Co(OH)_2_ particles were dispersed in 5 mL of ethanol under ultrasonic treatment for at least 20 min until it was mixed uniformly. Carbon nitride powders were dispersed and sonicated in 20 mL of ethanol for another 15 min, then the above Co(OH)_2_ suspension was added to the mixture. The mixture was dried in an oil bath at 80 °C overnight, in order to remove ethanol. Then, the uniform powders were annealed at 120 °C in a muffle furnace for 2 h, obtaining X% Fw-Co(OH)_2_/g-C_3_N_4_ composites. X% is the weight ratio of Fw-Co(OH)_2_. The materials of synthesis in addition to the degradation process are shown in Figure 1.

### 2.3. Characterization

X-ray diffraction (XRD) patterns were recorded on an X-ray diffractometer (Bruker, Bremen, Germany) with Cu-Kα radiation (λ = 1.5406 Å) over a 2θ angle range of 10–80°. X-ray photoelectron spectroscopy (XPS) were collected on a K-alpha X-ray photoelectron spectrometer (Thermo ESCALAB 250Xi, Carlsbad, CA, USA) with monochromatic Al Kα (hv = 1486.6 eV) from an X-ray source operating at 15 kV and 10 mA. All binding energies were referenced to the C 1s peak at 284.6 eV. The morphology of the photocatalysts were investigated via a scanning electron microscope (SEM, FEI Quanta 400 FEG), which was equipped with energy dispersive spectroscopy (EDS). High-resolution transmission electron microscopy (HRTEM) images were measured using a FEI Tecnai G2 F20 (HRTEM, FEI, Hillsboro, OR, USA), with an accelerating voltage of 200 kV. UV–visible diffuse reflectance spectra (UV–vis DRS) of the samples were measured on a UV–visible spectrophotometer (UV-2600, SHIMADZU, Kyoto, Japan) over the range of 200–800 nm using BaSO_4_ as a reference. The binding energy was calibrated with reference to the C1 s peak at 284.8 eV.

### 2.4. Photoelectrochemical Measurements

Photocurrents were measured by an electrochemical analyzer (PARSTAT-4000, Oak Ridge, TN, USA) in a standard three-electrode system, using a Pt sheet (2 × 2 cm^2^) as the counter electrode and saturated calomel electrode (SCE) as the reference electrode. Each conductive F-doped tin oxide (FTO) glass (effective area 2 × 2 cm^2^) was cleaned using sonication in ethanol for 30 min, dried at 80 °C and then coated with a thin layer of Fw-Co(OH)_2_/g-C_3_N_4_ catalyst as the substrate. The catalyst layer was prepared with 8 mg as-prepared sample powder (Fw-Co(OH)_2_/g-C_3_N_4_) in a solution of 50 μL of Nafion (5 wt%) and 25 mL of ethyl alcohol. The catalyst suspensions were transferred to the FTO glasses using a pipette and then dried at 60 °C. The loading of catalyst on the electrode was ≈2 mg/cm^2^. A 300-Watt xenon arc lamp (PLS-SXE300, Bofeilai Technology Co., Ltd., Beijing, China) with an excitation wavelength of 420 nm was used as the visible light source. A 0.1 M Na_2_SO_4_ aqueous solution was used as the electrolyte. For photocurrent measurement (I–t curves), the aforementioned 300-Watt Xe lamp was equipped with a 420 nm cut-off filter as the light source.

### 2.5. Photo(electro)catalytic Degradation

The catalytic activities of samples (prepared Fw-Co(OH)_2_/g-C_3_N_4_ catalyst loaded onto FTO glass substrates) were evaluated by photo(electro)catalytic decomposition of MB. The materials of the degradation process are shown in Figure 1. The photocatalysis test was carried out with a 100-milliliter solution of MB (0.1 mol/L) under visible irradiation and constant voltage. The light was obtained from a 300-Watt Xe lamp with a 420 nm cutoff filter. The distance between the samples and lamp was 10 cm, with a constant light intensity of 200 W. The constant voltage was provided by a voltage source of 3 V. Prior to irradiation, the fabricated 2.0 × 2.0 cm^2^ samples were immersed in the MB (100 mL, 0.1 M) solution that reacted with the catalyst electrode for 0.5 h in the dark at room temperature, in order to establish an adsorption/desorption equilibrium. The concentrations of MB in the sampled solutions were monitored by a UV–visible spectrophotometer (UV-2600, SHIMADZU) at its characteristic wavelength (k = 665 nm). The ratio of MB concentrations C/C_0_ could be calculated using C/C_0_ = A/A_0_, where C_0_ and C are the concentrations of MB solution at irradiation time 0 and t, respectively, and the respective A_0_ and A are the corresponding absorbance values at 665 nm. In the durability testing, three successive cycles were performed. After each cycle (V = 3 V, LI = 200 W), the photocatalysts were washed with ethanol and deionized water carefully, and then dried at 60 °C for 12 h. Then, the used photocatalysts were inserted into fresh 0.1 mol/L pollutant solution in order to carry out the next photoelectrochemical activity testing.

This paper evaluated MB degradation efficiency as a result of an applied electric field and photocatalyst; different parameters combinations were applied: (i) four different voltage values at the same light intensity (200 W) were specified: 0 V, 1 V, 2 V and 3 V, and (ii) four different light intensities at the same voltage (3 V) were employed: 0 W, 50 W, 100 W and 200 W. The light intensity was measured by the PL-MW2000.

## 3. Results

### 3.1. Characterization of Fw-Co(OH)_2_/g-C_3_N_4_

**Figure 2:**
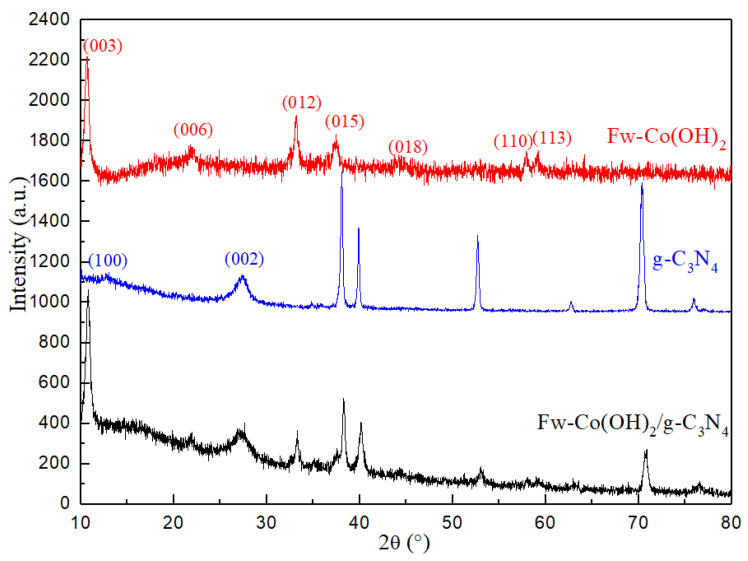
The XRD patterns of Fw-Co(OH)_2_, g-C_3_N_4_ and Fw-Co(OH)_2_/g-C_3_N_4_ composites.

It can be seen clearly that the (100) and (002) peaks of g-C_3_N_4_ are at 13.1° and 27.4°, respectively [25]. The XRD pattern of cobalt hydroxide display diffraction peaks at 2θ values of 10.5, 21.3, 33.2, 37.1, 57.9 and 59.4° (Figure 2), which could be indexed to (003), (006), (012), (015), (110) and (013), respectively; these reflect a hydrotalcite-like α-Co(OH)_2_ structure rather than the standard β-Co(OH)_2_ with a broad diffraction peak between about 20° and 30° [26,27]. The maximal diffraction peak exhibited two series of diffraction peaks, which can be indexed to the α-Co(OH)_2_ and g-C_3_N_4_, respectively. The hydrolysis reaction of urea with crystallized water in cobaltous acetate generates OH^-^ anions, which reacted with Co^2+^ to form Co(OH)_2_.

**Figure 3:**
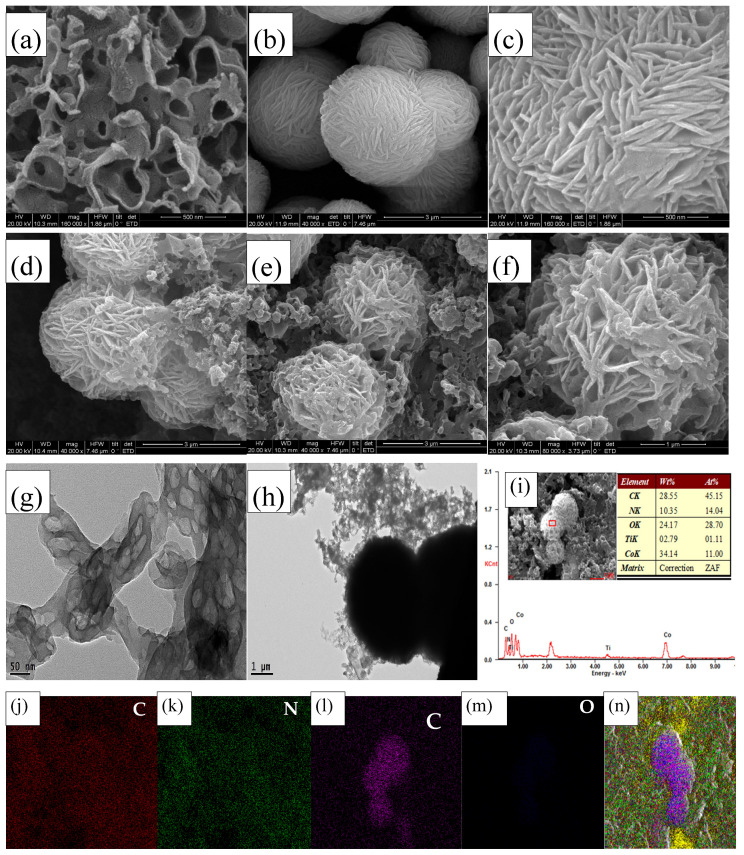
(**a**) SEM images of g-C_3_N_4_, (**b**,**c**) Fw-Co(OH)_2_, (**d**–**f**) Fw-Co(OH)_2_/g-C_3_N_4_; (**g**) TEM images of g-C_3_N_4_, (**h**) Fw-Co(OH)_2_/g-C_3_N_4_; (**i**) EDX of Fw-Co(OH)_2_/g-C_3_N_4_; (**j**–**n**) elemental mapping images of a Fw-Co(OH)_2_/g-C_3_N_4_ sample.

Figure 3a shows that the SEM images of the g-C_3_N_4_ nanosheets, which exhibit a layered morphology and a sheet-like structure. From the SEM images of Fw-Co(OH)_2_ in Figure 3b,c, it can be intuitively observed that the morphology of the samples are nanospheres around 2 μm in diameter. Detailed observations show that these 3D flower-like architectures are constructed by intertwined two-dimensional (2D) nanosheets with thicknesses of around 20 nm. According to the literature, with the assistance of PVP, the hydroxides are assembled into rod and then flake precipitates which stack and form flower-like architectures. As seen in Figure 3d–f, an SEM image of the boundary of the Fw-Co(OH)_2_ nanoparticles is displayed in g-C_3_N_4_, indicating the formation of Fw-Co(OH)_2_/g-C_3_N_4_ heterostructures in as-prepared 25% Fw-Co(OH)_2_/g-C_3_N_4_ samples (the weight ratio of Fw-Co(OH)_2_ and Fw-Co(OH)_2_ is 1:3) [11]. The combined nanosheet structure of g-C_3_N_4_ and Fw-Co(OH)_2_ not only provides abundant adsorption sites for the MB molecules, but also greatly shortens the transport distance of photogenerated charge carriers. TEM image shown in Figure 3g,h, was used to further explore the detailed structure of the g-C_3_N_4_ and Fw-Co(OH)_2_/g-C_3_N_4_ sample, and indicated the formation of Fw-Co(OH)_2_/g-C_3_N_4_ heterostructures in as-prepared 25% Fw-Co(OH)_2_/g-C_3_N_4_ samples. Elemental mapping of the Fw-Co(OH)_2_/g-C_3_N_4_ microparticles recorded using energy dispersive spectroscopy are shown in Figure 3j–n), where red, green, purple and black refer to C, N, Co and O elements, respectively, indicating that the cobalt hydroxide compound was distributed over the surface of the g-C_3_N_4_ base material. EDX results, as shown in Figure 3i, prove that the weight ratios of Co and C in both flower-like 25% Fw-Co(OH)_2_/g-C_3_N_4_ nanostructures are both 1.1:1, which are consistent with the preparation conditions.

**Figure 4:**
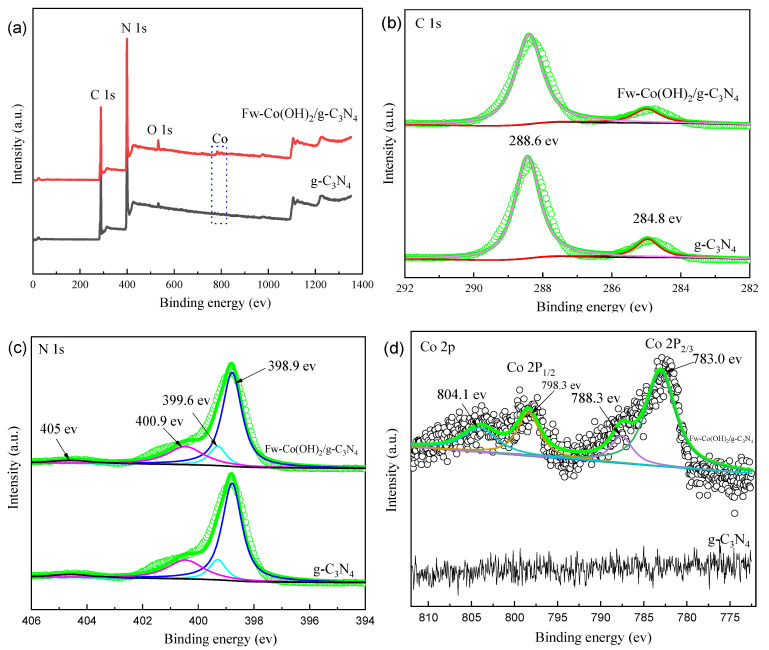
(**a**) XPS survey spectra of g-C_3_N_4_ and Fw-Co(OH)_2_/g-C_3_N_4_; XPS spectra of g-C_3_N_4_ and Fw-Co(OH)_2_/g-C_3_N_4_: (**b**) C 1s; (**c**) N 1s; and (**d**) Co 2p.

X-ray photoelectron spectroscopy analysis was employed to reveal the surface chemical composition and the detailed electronic states of different elements in g-C_3_N_4_ and in Fw-Co (OH)_2_/g-C_3_N_4_ composites. The XPS survey spectra (Figure 4a) suggests that the Fw-Co(OH)_2_/g-C_3_N_4_ was primarily composed of C, N, O and Co, further confirming the co-existence of g-C_3_N_4_ and Fw-Co (OH)_2_/g-C_3_N_4_ in the composite. The C1 s spectra (Figure 4b) showed two deconvoluted peaks at 288.6 eV and 284.8 eV, ascribed to the signals of sp2-bonded carbon (N-C=N) and standard reference carbon, which is usually observed in the XPS spectra of carbon nitrides [28]. In addition, the N 1s spectrum in Figure 4c can be deconvoluted into four peaks located at 398.9 eV, 399.6 eV, 400.9 eV and 405 eV, which can be assigned to C=N-C, (C)_3_-N, quaternary N bonded to three carbon atoms in the aromatic cycles, and the π citations, respectively [17,29]. In conjunction with the above results, except for the peaks’ intensities, the bonding characteristics of C1 s and N1 s showed no apparent change between g-C_3_N_4_ and Fw-Co (OH)_2_/g-C_3_N_4_. The existence of Co (OH)_2_ can be further certified by high resolution XPS analysis of Co 2p. In Figure 4d, the high XPS resolution of Co 2p can be deconvoluted into two pairs of individual peaks at 783.0 eV and 798.3 eV, respectively, which were identified as the major binding energies of Co^2+^ in Co (OH)_2_ [20].

### 3.2. Optical Studies

**Figure 5:**
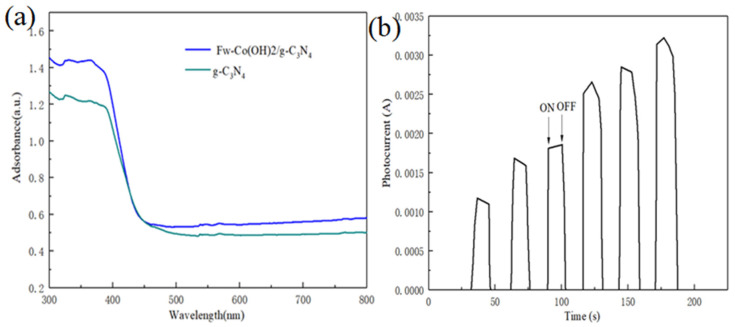
(**a**) UV–vis diffuse reflectance spectra of Fw-Co(OH)_2_/g-C_3_N_4_ and g-C_3_N_4_; (**b**) transient photocurrent responses of the Fw-Co(OH)_2_/g-C_3_N_4_ sample in 1M Na_2_SO_4_ aqueous solution under visible-light irradiation at 0.5 V vs. Ag/AgCl.

The UV–vis diffuse reflectance spectra of as-prepared samples are shown in Figure 5. As seen in Figure 5a, the as-prepared samples exhibit an obvious adsorption edge at about 445 nm, corresponding to a band gap of 2.78 eV. Clearly, the absorption edges of Fw-Co(OH)_2_/g-C_3_N_4_ were shifted to a longer-wavelength region than that of pure g-C_3_N_4_. Moreover, there was an increase in optical absorption in wavelengths ranging from 430 to 750 nm after loading Fw-Co(OH)_2_ onto g-C_3_N_4_. This result shows that addition of Fw-Co(OH)_2_ enhances light-harvesting ability of Fw-Co(OH)_2_/g-C_3_N_4_ in the visible-light region. Notably, compared with the edge of g-C_3_N_4_, the shape of Fw-Co(OH)_2_/g-C_3_N_4_ did not alter, indicating that the loading of Fw-Co(OH)_2_ had no effect on the lattice of g-C_3_N_4_.

Figure 5b shows the I–t curves for the 25% Fw-Co(OH)_2_/g-C_3_N_4_ with several on-off cycles of intermittent irradiation. As can be seen from this figure’s data, the photocurrent value rapidly decreased to zero as soon as the irradiation was turned off, and the photocurrent came back to a constant value when the light was turned on again. This indicates that most of the photogenerated electrons were transported to the back contact across the samples to produce photocurrent under visible-light irradiation.

### 3.3. Photo(electro)catalytic Degradation of Methylene Blue

**Figure 6:**
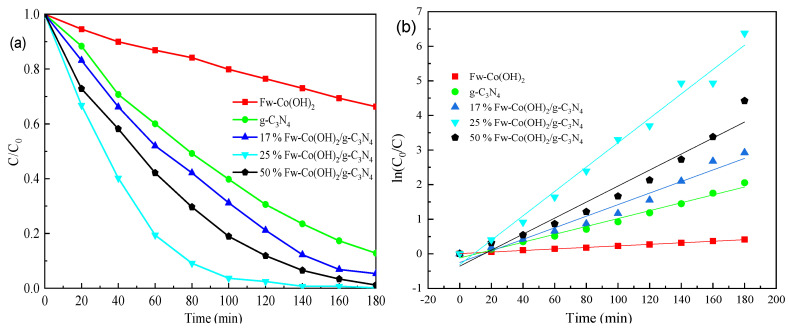
(**a**) Photo(electro) degradation of MB, (**b**) linear transform ln (C_0_/C) of the kinetic curves of MB using different catalysts (light intensity in visible light = 200 W, applied voltage = 200 W).

In order to optimize the degradation of MB, different ratios of Fw-Co(OH)_2_ (17, 25 and 50 wt.%) were loaded onto g-C_3_N_4_. Figure 6 displayed that, with the weight ratio of Fw-Co(OH)_2_ increased to 25%, degradation significantly increased also. With a ratio of 25% Fw-Co(OH)_2_/g-C_3_N_4_, degradation can achieve about a 100% ratio within 180 min using visible light, which compared by 17% Fw-Co(OH)_2_/g-C_3_N_4_ that degraded 65% and 50% Fw-Co(OH)_2_/g-C_3_N_4_ that degraded with 80% efficiency. When the weight ratio was further elevated to 50%, the rate dropped by 20%, possibly as a result of extravagant loading of Fw-Co(OH)_2_ that blocked the optical absorption of g-C_3_N_4_. Moreover, MB dye (0.1 moL/L) was degraded by about 32%, 83% and almost 100% for Fw-Co(OH)_2_, g-C_3_N_4_ and Fw-Co(OH)_2_/g-C_3_N_4_ nanocomposites, respectively, after 180 min. It can be easily observed that the Fw-Co(OH)_2_/g-C_3_N_4_ composites showed remarkably enhanced performance compared to pure g-C_3_N_4_ and Fw-Co(OH)_2_. These results suggest that the synergistic and intimate interaction effects between Fw-Co(OH)_2_ and g-C_3_N_4_ are crucial for visible-light catalytic activity. The pseudo-first order equation specified below was applied in order to extract the reaction kinetics of the MB:

(1) $$\ln\left(\frac{C_{0}}{C}\right)=\mathrm{kt}$$

where C is the concentration of the dye at time t, C_0_ is concentration at time 0 and k is the pseudo-first order rate constant. The plots of ln (C_0_/C) against irradiation time drawn for all the samples are shown in Figure 6. The degradation rate constants (k) were calculated from the slopes of straight lines drawn using linear regression, according to the first-order kinetic law ln(C_0_/C) = kt [30,31]. The calculated k values and the corresponding linear regression coefficient degree (R^2^) values are shown in Table 1. The values of k and R^2^ also clearly indicate that the 25% Fw-Co(OH)_2_/g-C_3_N_4_ preparation exhibits better photocatalytic performance. That is, an appropriate Fw-Co(OH)_2_ amount promotes photocatalytic activity of g-C_3_N_4_ through preventing the recombination of electron-hole pairs. However, abundant loading may possibly hinder the recombination center of photo-generated charge carriers [11].

**Figure 7:**
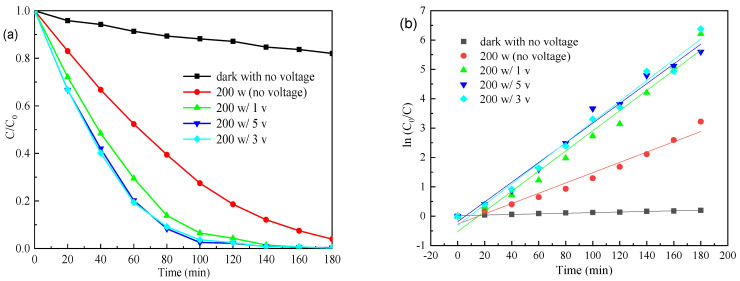
(**a**) Photo (electro) degradation of MB, (**b**) linear transform ln (C_0_/C) of the kinetic curves of MB by 25% Fw-Co(OH)_2_/g-C_3_N_4_ at different voltages.

**Figure 8:**
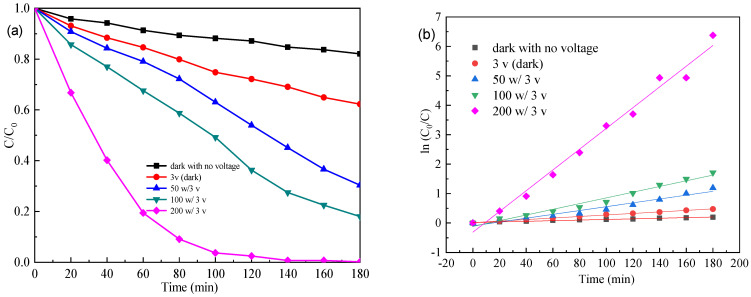
(**a**) Photo (electro) degradation of MB, (**b**) linear transform ln (C_0_/C) of the kinetic curves of MB by 25% Fw-Co(OH)_2/_g-C_3_N_4_ at different visible light intensities.

Figure 7 and Figure 8 show the effects of the visible irradiation intensity and applied voltage on the photo (electro) degradation performances of the 25% Fw-Co(OH)_2_/g-C_3_N_4_ in MB solution, respectively. As seen in Figure 7, when the applied voltage increases from 1 V to 5 V, MB removal was only slightly higher. Especially when the voltage changes from 3 V to 5 V, decolorization of MB was achieved to nearly the same extent. The degradation of MB obeyed the pseudo-first-order kinetic model. The apparent rate constants k was summarized in Table 1. The values of MB degradation at different applied voltages had a little difference. As shown in Figure 8, photodegradation of dyes changes with the intensity of light. The k values of MB degradation follow in this order: 200 W > 100 W > 50 W > 0 W (dark). The photocatalytic activity of 25% Fw-Co(OH)_2_/g-C_3_N_4_ drastically increase with increasing light intensity, from 0 W to 200 W. Results reveal that changes in light intensity (50 W to 200 W) play a more important role than the voltage in this system.

### 3.4. Photocatalytic Degradation Mechanism

**Figure 9:**
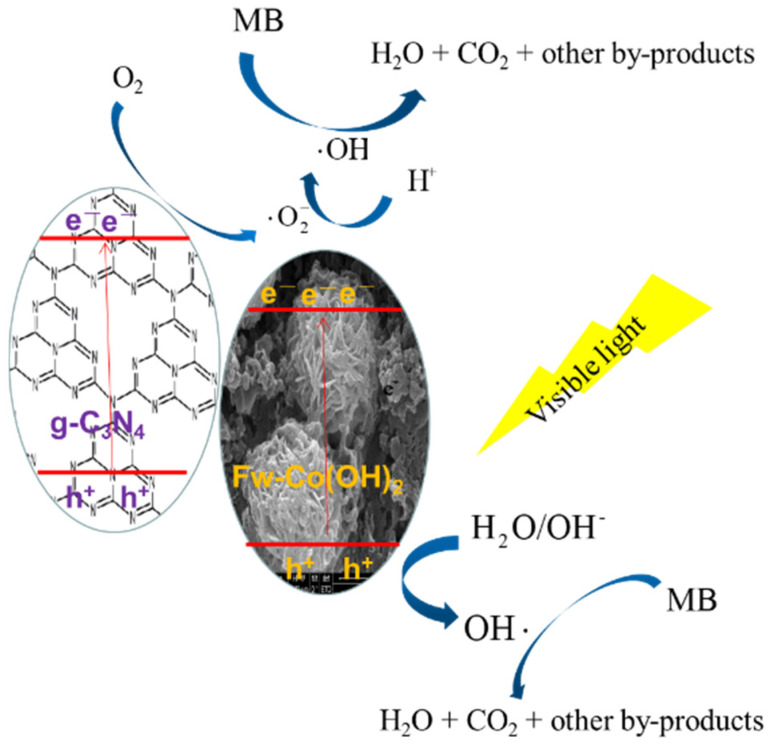
Schematic illustration of a possible mechanism of Fw-Co (OH)_2/_g-C_3_N_4_ in MB photocatalytic degradation.

Under visible light irradiation, an electron-hole pair forms, and then a conduction-band electron and a valence-band hole separate on the surface of Fw-Co(OH)_2_/g-C_3_N_4_ (seen in Figure 9). Perhaps the separated hole permits the direct oxidation of MB to reactive intermediates. The increased photocatalytic activity of the coupled photocatalyst was primarily attributed to the enhancements in charge separation efficiency, since a junction was formed by the decoration of Fw-Co(OH)_2_ with g-C_3_N_4_. The PL spectra results also prove that after loading Co(OH)_2_ NPs onto the surface of g-C_3_N_4_, the average lifetimes were significantly increased [32]. Co(OH)_2_ NP loading remarkably reduced the recombination of carriers, and prolonged the lifetime of photogenerated charge. The junction played an important role in the separation of photogenerated electron-hole pairs. Upon visible excitation, the photogenerated electrons of the Fw-Co(OH)_2_ conduction band are transferred to the conduction band of g-C_3_N_4_. Since the holes move in the opposite direction from the electrons, photogenerated holes become trapped within the Fw-Co(OH)_2_ particle, causing charge separation to become more efficient. The photogenerated electrons accumulate on the surface of g-C_3_N_4_, and can be rapidly transferred to molecular oxygen O_2_ to form the superoxide radical anion·O_2_^−^, which could subsequently generate ·OH radicals and act as effective centers of organic matter mineralization for photocatalytic reactions. The positive holes in the valence band could be trapped by OH^−^ or H_2_O species adsorbed on the surface of the catalyst, producing reactive hydroxyl radicals in aqueous media. The active oxygen vacancies that severed as electron acceptors trapped the photoinduced electrons temporarily to restrain the surface recombination of photogenerated electrons and holes. Thus, photocatalytic systems provide broad-range visible light absorption in addition to efficient space separation of photogenerated charge carriers, resulting in efficacious photoactivity. The mechanistic insights infer photocarrier migration and separation, along with the generation of reactive radical species involved in the photodegradation process [11].

### 3.5. Stability and Reusability

**Figure 10:**
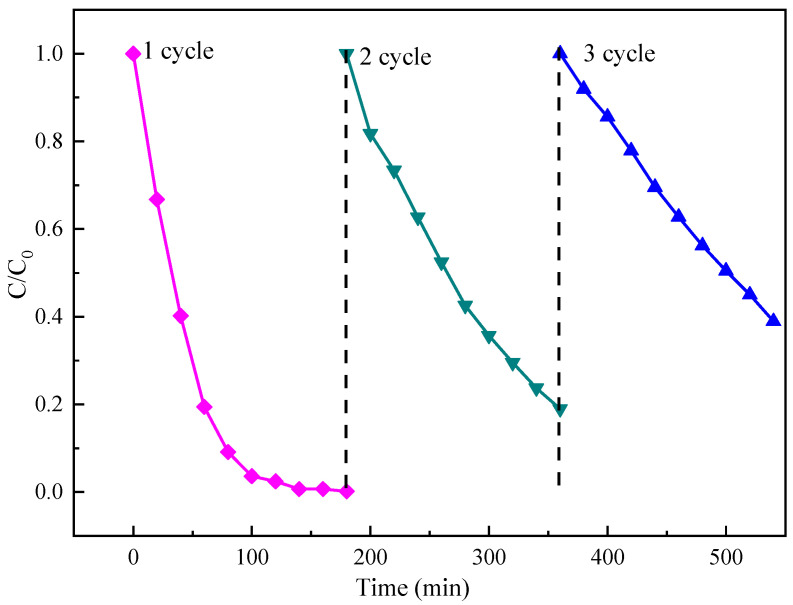
Cyclic photocatalysis of MB under visible light.

Practical applicability, stability and recyclability all play an important role in the photocatalytic degradation process. The used Fw-Co(OH)_2_/g-C_3_N_4_ catalyst was soaked in deionized water for ten minutes, separated by centrifugation, dried at 60 °C under vacuum and reused. Results in Figure 10 show photocatalytic MB degradation during three consecutive runs with reused photocatalyst. As seen in Figure 10, the stability of 25% Fw-Co(OH)_2_/g-C_3_N_4_ catalyst was evaluated; it achieved about 83% and 64% photocatalytic degradation efficiency when the Fw-Co(OH)_2_/g-C_3_N_4_ catalyst was reused for the second and third times, respectively. The results show that these materials maintain high MB dye degradation capability after three consecutive cycles under identical conditions, which indicates long-term stability of Fw-Co(OH)_2_/g-C_3_N_4_. The decrease in degradation efficiency may be related to the loss of catalyst during the repeated reuse process. Thus, it could be concluded that Fw-Co(OH)_2_/g-C_3_N_4_ nanocomposite exhibits good stability and recyclability during the photocatalytic process. This nanocomposite can provide a technical foundation for the use of non-metallic catalysts in dye wastewater treatment.

## 4. Conclusions

In summary, Fw-Co(OH)_2_/g-C_3_N_4_ nanocomposite was synthesized via a hydrothermal method for testing enhancement of the photo(electro)catalytic degradation effect. The research results demonstrate that Fw-Co(OH)_2_/g-C_3_N_4_ nanocomposite exhibits better photocatalytic activity as compared to the Fw-Co(OH)_2_ and g-C_3_N_4_ alone, which was mainly attributed to the synergistic effects of interfaces between Co(OH)_2_ and g-C_3_N_4_, and to low recombination of the photogenerated electrons and holes. These nanomaterials were characterized for structure, surface morphology and composition using XRD, SEM and XPS, respectively. The photocatalysts based on g-C_3_N_4_ proved their worth by providing extend visible-light absorption, improved space charge separation, along with stimulated migration and enhanced efficacy of various photocatalytic activities, all consistent with previous research [11]. Moreover, the prepared nanocomposite (at the weight ratio of Fw-Co(OH)_2_ to g-C_3_N_4_ equaling 1:3) exhibited excellent photocatalytic response by completely degrading the MB dye at 180 min in a standard three-electrode system. In addition, the Fw-Co(OH)_2_/g-C_3_N_4_ nanocomposite demonstrated that it has good stability for photocatalytic degradation even after three repeated uses. Finally, a suitable photocatalytic reaction mechanism has been proposed to explain the photocatalytic performance of the nanocomposite. The Fw-Co(OH)_2_/g-C_3_N_4_ nanocomposite has superior activity and physico-chemical stability, it can provide a technical foundation for the use of non-metallic catalysts, and has good prospects for becoming widely used in dye wastewater treatment.

## Figures and Tables

**Table 1:** The apparent pseudo-first-order rate parameters of Fw-Co(OH)_2_/g-C_3_N_4._

Sample	Light Intensity (W)	Voltage (V)	Rate Constant (K), min^−1^	R^2^
Fw-Co(OH)_2_	200	3	0.0022 × 10^−5^	0.9976
g-C_3_N_4_	200	3	0.0114 × 10^−4^	0.9871
17% Fw-Co(OH)_2_/g-C_3_N_4_	200	3	0.0232	0.9540
25% Fw-Co(OH)_2_/g-C_3_N_4_	200	3	0.0353	0.9856
50% Fw-Co(OH)_2_/g-C_3_N_4_	200	3	0.0167	0.9656
25% Fw-Co(OH)_2_/g-C_3_N_4_	—	—	0.0010 × 10^−5^	0.9801
25% Fw-Co(OH)_2_/g-C_3_N_4_	200	—	0.0175	0.9689
25% Fw-Co(OH)_2_/g-C_3_N_4_	200	1	0.0343	0.9736
25% Fw-Co(OH)_2_/g-C_3_N_4_	200	5	0.0336	0.9849
25% Fw-Co(OH)_2_/g-C_3_N_4_	—	3	0.0026 × 10^−5^	0.9970
25% Fw-Co(OH)_2_/g-C_3_N_4_	50	3	0.0065 × 10^−4^	0.9807
25% Fw-Co(OH)_2_/g-C_3_N_4_	100	3	0.0097 × 10^−4^	0.9761

## Data Availability

All data generated or analyzed during the current study are included in this published article.
